# Perioperative management of a patient with severe aortic stenosis and chronic obstructive pulmonary disease undergoing abdominal aortic aneurysm repair: A case report

**DOI:** 10.1016/j.ijscr.2025.110871

**Published:** 2025-01-13

**Authors:** Ibrahim Nagmeldin Hassan, Siddig Yaqub, Mohamed Ibrahim

**Affiliations:** a University of Khartoum, Faculty of Medicine, Khartoum, Sudan; b Karary University, Faculty of Medicine, Khartoum, Sudan; c Al-Neelain University, Faculty of Medicine, Khartoum, Sudan

**Keywords:** Severe aortic stenosis, COPD, Perioperative management, Abdominal aortic aneurysm, Multidisciplinary care

## Abstract

**Introduction and Importance:**

Severe aortic stenosis (AS) and chronic obstructive pulmonary disease (COPD) significantly increase perioperative morbidity and mortality. This case report discusses the challenges of managing a 75-year-old male patient with severe AS and advanced COPD undergoing elective abdominal aortic aneurysm (AAA) repair.

**Case presentation:**

The patient presented with a 6.5 cm infrarenal AAA identified during routine surveillance. His medical history included severe AS (valve area 0.7 cm^2^) and advanced COPD (FEV1 45 % of predicted). A multidisciplinary team developed a management plan that included pulmonary rehabilitation and cautious medication use. On the day of surgery, general anaesthesia was induced, with invasive haemodynamic monitoring established to maintain stability. Norepinephrine was titrated to achieve a mean arterial pressure of 70–80 mmHg, while fluid management was carefully monitored to prevent volume overload. The AAA repair was completed successfully without major complications.

**Clinical discussion:**

This case highlights the complexities of managing patients with severe AS and COPD during major surgery. Effective fluid management and lung-protective ventilation strategies minimized respiratory complications. Postoperatively, non-invasive ventilation facilitated early extubation and improved respiratory function.

**Conclusion:**

This case underscores the importance of a multidisciplinary approach in managing complex patients with significant comorbidities. Collaboration among specialists is vital to optimize outcomes in patients undergoing major surgical interventions.

## Highlights

• Multidisciplinary care is key in AAA repair with severe aortic stenosis and COPD.
• Intraoperative fluid control and monitoring ensure haemodynamic stability.
• Postoperative respiratory support prevents cardiac and pulmonary complications.

## 1 Introduction

Severe aortic stenosis (AS) and chronic obstructive pulmonary disease (COPD) are significant risk factors for perioperative morbidity and mortality in surgical patients [1]. AS poses challenges for maintaining haemodynamic stability, while COPD increases the risk of respiratory failure [2]. Managing patients with both conditions during major surgery presents a considerable challenge. In this case, transcatheter aortic valve replacement (TAVR) was considered as a potential intervention for the severe AS. However, it was deferred due to the urgency of the abdominal aortic aneurysm (AAA) repair, which was deemed life-threatening if delayed further. This decision was guided by a multidisciplinary team's assessment of risks and benefits, taking into account the patient's comorbidities and current clinical stability.

This case report describes the perioperative management of a 75-year-old male with severe AS and advanced COPD undergoing elective abdominal aortic aneurysm (AAA) repair.

## 2 Case presentation

A 75-year-old male presented with a 6.5 cm infrarenal abdominal aortic aneurysm, discovered during routine surveillance. His past medical history was significant for severe AS (valve area of 0.7 cm^2^, mean gradient of 50 mmHg) and advanced COPD (FEV1 45 % of predicted). The patient reported progressive dyspnoea on exertion but denied chest pain or syncope. He was maintained on inhaled bronchodilators (tiotropium 18 μg once daily, salbutamol as needed) and corticosteroids (fluticasone 500 μg twice daily). Given his severe comorbidities, a multidisciplinary team was assembled, including cardiology, pulmonology, anaesthesia, and vascular surgery. Although less invasive approaches, such as endovascular aneurysm repair (EVAR), were considered for the AAA repair, an open surgical approach was selected due to anatomic considerations and the patient's overall clinical presentation. Staged procedures, such as addressing the aortic stenosis prior to AAA repair, were also evaluated but were deemed inappropriate given the immediate risk posed by the aneurysm.

Pulmonary rehabilitation was initiated to improve lung function, and the patient was prescribed oral prednisolone 30 mg daily for 5 days before surgery to reduce the risk of postoperative respiratory complications. Beta-blockers were avoided to prevent bronchospasm due to his COPD. At the time of admission, his vital signs showed a blood pressure of 135/75 mmHg, a heart rate of 80 beats per minute, a respiratory rate of 18 breaths per minute, and an oxygen saturation of 94 % on room air. Echocardiography confirmed an ejection fraction (EF) of 55 %.

On the day of surgery, general anaesthesia was induced with propofol 2 mg/kg, fentanyl 2 μg/kg, and rocuronium 0.6 mg/kg to facilitate endotracheal intubation. Maintenance of anaesthesia was achieved with sevoflurane 1.5 % and an infusion of remifentanil at 0.1 μg/kg/min. Given the severe AS, invasive haemodynamic monitoring was established, including a radial arterial line and a pulmonary artery catheter. Continuous cardiac output and mixed venous oxygen saturation were measured throughout the procedure. Initial intraoperative measurements included a blood pressure of 125/70 mmHg, a heart rate of 85 beats per minute, a central venous pressure (CVP) of 10 mmHg, a cardiac output of 4.5 L/min, and a systemic vascular resistance (SVR) of 1500 dynes/s/cm^5^.

To maintain haemodynamic stability, an infusion of norepinephrine was titrated, starting at 0.05 μg/kg/min and increased to 0.1 μg/kg/min as needed to maintain a target mean arterial pressure (MAP) of 70–80 mmHg, avoiding hypotension that could compromise coronary perfusion. Fluid administration was guided by stroke volume variation and CVP, with 500 mL of lactated Ringer's solution infused over the first 2 h, followed by additional boluses as needed. Care was taken to avoid excessive fluid shifts, as these could increase afterload and compromise left ventricular function. For ventilation, lung-protective strategies were employed. Tidal volumes were set at 6 mL/kg of ideal body weight (450 mL), with positive end-expiratory pressure (PEEP) of 5 cmH₂O, and peak airway pressures were kept below 30 cmH₂O. Arterial blood gas analysis intraoperatively revealed a pH of 7.35, PaCO₂ of 48 mmHg, PaO₂ of 100 mmHg on FiO₂ of 0.5, and HCO₃^−^ of 25 mEq/L. The abdominal aortic aneurysm was successfully repaired over a period of 4 h without major complications. The patient remained haemodynamically stable throughout, with norepinephrine titrated between 0.05 and 0.1 μg/kg/min.

Postoperatively, the patient was transferred to the intensive care unit (ICU) for continued monitoring. He remained intubated and sedated with propofol at 50 μg/kg/min and remifentanil at 0.05 μg/kg/min infusions. Haemodynamic parameters remained stable, with a blood pressure of 130/75 mmHg, a heart rate of 80 beats per minute, and a CVP of 12 mmHg. During the first 24 h postoperatively, the patient developed mild pulmonary oedema, likely due to fluid shifts and his underlying cardiac condition. Diuresis was initiated with intravenous furosemide 40 mg, which improved oxygenation and reduced fluid overload. Daily fluid balance was carefully monitored. The patient was weaned from mechanical ventilation over the next 48 h. Extubation was performed once the arterial blood gas showed improved respiratory function with a pH of 7.40, PaCO₂ of 44 mmHg, and PaO₂ of 98 mmHg on FiO₂ of 0.4. Post-extubation, the patient was placed on non-invasive ventilation (BiPAP) with inspiratory positive airway pressure (IPAP) of 12 cmH₂O and expiratory positive airway pressure (EPAP) of 6 cmH₂O to support respiratory function and prevent atelectasis. Supplemental oxygen was provided via nasal cannula at 2–3 L/min to maintain oxygen saturation above 92 %. Over the subsequent days, the patient was transitioned to room air with no further pulmonary or cardiac complications. He was discharged from the ICU on postoperative day 5 and continued to recover well on the vascular ward.

## 3 Discussion

This case highlights the challenges of perioperative management in a patient with both severe aortic stenosis and chronic obstructive pulmonary disease undergoing major vascular surgery. In patients with severe AS, maintenance of haemodynamic stability is essential, as rapid fluctuations in blood pressure can lead to decreased coronary perfusion and cardiac ischaemia. Norepinephrine was titrated carefully to maintain a MAP between 70 and 80 mmHg, which ensured adequate perfusion without excessive increases in afterload [3].

Fluid management was another critical aspect of care. Patients with AS require careful monitoring of fluid status to avoid both volume overload and under-resuscitation. In this case, stroke volume variation and CVP were used to guide fluid administration, and small fluid boluses were given cautiously to avoid excessive preload increases [4].

Respiratory management was equally challenging due to the patient's advanced COPD. Lung-protective ventilation with low tidal volumes and moderate PEEP minimized the risk of barotrauma while supporting gas exchange. The postoperative use of non-invasive ventilation (BiPAP) helped prevent atelectasis and facilitated early weaning from mechanical ventilation, avoiding prolonged intubation, which is particularly risky in COPD patients [2].

This case demonstrated the critical need for close adherence to current evidence-based guidelines when managing patients with peripheral arterial disease and significant comorbidities. The 2024 European Society of Cardiology (ESC) guidelines for peripheral arterial disease and aortic aneurysms recommend a multidisciplinary approach, careful perioperative haemodynamic management, and individualized care plans, all of which were central to this patient's successful outcome [5].

Follow-up after discharge revealed that the patient remained asymptomatic during the first three months, with no further respiratory or cardiac complications reported. Routine vascular and cardiac outpatient follow-ups were scheduled to monitor the repaired AAA and severe AS, respectively. This emphasizes the importance of continued multidisciplinary care in optimizing long-term outcomes.

## 4 Conclusion

This case underscores the importance of a multidisciplinary approach in managing complex patients with significant comorbidities. Involving specialists from cardiology, pulmonology, anaesthesia, and vascular surgery ensured that both the cardiac and respiratory needs of the patient were addressed preoperatively, intraoperatively, and postoperatively [6]. This case report was written in accordance with the SCARE 2023 criteria (Surgical CAse REport guidelines) to ensure proper structure, reporting standards, and quality for surgical case reports [7].

## Consent

Written informed consent was obtained from the patient for publication. A copy of the written consent is available for review by the Editor-in-Chief of this journal on request.

## Ethical approval

Exemption from ethical approval has been granted by the Institutional Review Board (IRB) at our institution for this case report. Case reports do not require IRB approval according to the guidelines of our institution.

## Guarantor

Ibrahim Nagmeldin Hassan, University of Khartoum, Faculty of Medicine, Khartoum, Sudan

## Author contributions

I.N.H: conceptualization, data curation, methodology, and writing original draft;S.Y: writing, and revision of the original draft;M.I: methodology, investigation, writing, and revision of the original draft. All authors read and approved the final manuscript.

## Funding

None declared.

## Declaration of competing interest

None declared.
